# Advancing Sustainability in Hydrocarbon Production: Breakthroughs in CO_2_ Hydrogenation with Iron-Based Catalysts and Comprehensive Life Cycle Assessment of Environmental Impacts

**DOI:** 10.1021/acs.iecr.5c05039

**Published:** 2026-02-10

**Authors:** Arian Grainca, Veronica Bortolotto, Serena Biella, Alessandro Di Michele, Morena Nocchetti, Carlo Pirola

**Affiliations:** † 9304 Università Degli Studi di Milano, Dipartimento di Chimica, Via Golgi 19, Milano 20133, Italy; ‡ 225037 Università Degli Studi di Perugia, Dipartimento di Fisica E Geologia, Via Pascoli, Perugia 06123, Italy; § 9309 Università Degli Studi di Perugia, Dipartimento di Scienze Farmaceutiche, Via Del Liceo, Perugia 06123, Italy; ∥ Consorzio INSTM, Via G. Giusti 9, Firenze 50121, Italy

## Abstract

The need for carbon-neutral synthetic fuels drives research into CO_2_ hydrogenation via Fischer–Tropsch (FT) synthesis, where catalyst selection affects conversion efficiency and environmental performance. This study applies life cycle assessment to three hydrotalcite-derived catalysts (Fe30, Fe40, Co45), evaluating CO_2_ utilization efficiency, energy demand, and environmental impacts under laboratory-scale FT conditions. The CO_2_ utilization factor (CUF), defined as the ratio of CO_2_ consumed to emitted, reached 167% for Co45 at 350 °C, indicating net CO_2_ consumption despite burdens from cobalt production and critical raw material use. Iron-based catalysts offer lower production-related emissions but lower CO_2_ conversion, with Fe40 performing least favorably. Scenario analysis highlights electricity supply effects: replacing fossil power with hydro or biomass electricity improves CO_2_ sequestration but introduces land-use and ecotoxicity challenges. These findings expose limitations of extrapolating laboratory-scale LCA to industrial systems and support the development of carbon-negative FT fuels by guiding catalyst design, process efficiency, and energy integration.

## Graphical abstract

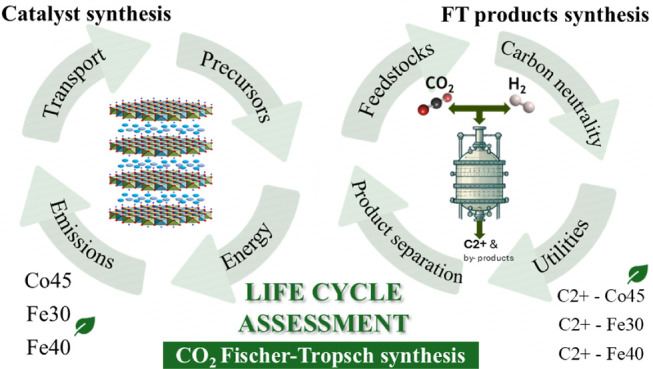

## 1 Introduction

Climate change is one of the most pressing global challenges of our time. The increase in atmospheric carbon dioxide (CO_2_) concentrations, primarily due to the use of fossil fuels, is a key factor contributing to the greenhouse effect and global warming.^1^ Projections indicate that, without significant interventions, global temperatures will continue to rise, leading to extreme weather events, sea-level rise, and biodiversity loss.^2^ In this context, reducing CO_2_ emissions has become an urgent priority to mitigate the effects of climate change.

Converting CO_2_ into useful products is a key mitigation strategy, and CO_2_ conversion technologies employ various chemical and catalytic processes, including hydrogenation, esterification, oxidation/reduction, and reverse water–gas shift reaction, as reviewed by Saravanan et al.,^3^ to produce valuable chemical compounds, such as carbonaceous fuels, polymers, and other building block chemicals.^4^ In this context, CO_2_ conversion technologies can be broadly classified into biological, electrochemical, and chemical routes, each with distinct advantages and limitations. Biological approaches use microorganisms to convert CO_2_ via photosynthesis or other metabolic pathways, and metabolic/bioengineering strategies have been explored to produce fuels and value-added chemicals.^5^ Other microbial routes can convert CO_2_ into energy carriers such as methane, although performance is strongly dependent on operating conditions.^6^ Electrochemical conversion technologies use electricity, typically from renewable sources, to reduce CO_2_ into chemicals such as formic acid, methanol, and ethylene using catalysts and operating under mild conditions. However, efficiency and selectivity remain challenging to be addressed at a large scale.^7^ Chemical conversion technologies involve catalytic processes to produce useful chemicals and fuels. One notable example is the Fischer–Tropsch (FT) reaction, which converts a mixture of carbon monoxide (CO) and hydrogen (H_2_) into liquid hydrocarbons. Other relevant routes include methanol synthesis from CO_2_/H_2_, the reverse water–gas shift (RWGS) reaction to generate CO, and dry reforming of methane (DRM) to produce syngas (CO and H_2_).^8^ These methods support efforts in carbon recycling and sustainable fuel production, as reflected in studies ranging from foundational mappings of plausible CO_2_ conversion reactions and target products^9^ to sustainability and life-cycle assessments of CO_2_ utilization routes^10^ and computational approaches guiding catalyst and process design.^11^

Among these technologies, CO_2_ hydrogenation coupled with reverse water–gas shift and Fischer–Tropsch synthesis is widely investigated for the production of liquid hydrocarbons from a CO_2_/H_2_ feed. When coupled with renewable hydrogen, for instance, produced by water electrolysis powered by wind or solar electricity, this route can contribute to more sustainable fuel production.^12^ The combination of CO_2_ capture and utilization with green H_2_ production thus represents an integrated approach to tackling the climate crisis and promoting energy sustainability.^13^

Lately, scientific research has focused on RWGS–FT routes starting from captured CO_2_ to produce high-quality FT fuels, such as diesel that can meet stringent specifications in various countries.^14^ In contrast to the classical Fischer–Tropsch process, the CO_2_-based process involves two distinct reactions when using an H_2_–CO_2_ mixture as input: the Reverse Water–Gas Shift reaction (eq 1) and the subsequent Fischer–Tropsch synthesis (eq 2). These reactions are endothermic and exothermic, respectively:

1 $${\mathrm{CO}}_{2}+H_{2}\to \mathrm{CO}+H_{2}O\;{\Delta H^{{}^{\circ}}}_{573K}=38{\mathrm{kJ mol}}^{\mathrm{‐1}}$$

2 $$\mathrm{CO}+2H_{2}\to -{\mathrm{CH}}_{2}-+H_{2}O\;{\Delta H^{{}^{\circ}}}_{573K}=-166{\mathrm{kJ mol}}^{\mathrm{‐1}}$$

The importance of the FT reaction lies in its ability to produce high-quality fuels that are compatible with existing infrastructure and engines, reducing the need for drastic changes in fuel distribution and consumption systems. Additionally, when paired with renewable hydrogen sources, such as those derived from water electrolysis using solar or wind energy, FT process can produce carbon-neutral or even carbon-negative fuels, significantly contributing to the reduction of greenhouse gas emissions.^15^

Catalysts, typically based on iron or cobalt, are essential to the FT reaction, as they influence the efficiency and selectivity of hydrocarbon production. Recent advancements in catalyst development, including iron-based hydrotalcite catalysts, have shown improved performance and stability, enhancing the viability of the FT process for sustainable fuel production.^16^ Double- and triple-layered hydroxides, also known as hydrotalcite-like compounds (HTlc), were initially studied in classical CO Fischer–Tropsch synthesis with promising results,^17^ prompting their evaluation in CO_2_ hydrogenation as well. HTlc are primarily composed of metal hydroxides, where specific metal atoms are uniformly dispersed at the atomic level. The general formula for HTlc is [M­(II)_1–*x*_M­(III)_*x*_(OH)_2_]^x+^(A^n–^_x/n_)^x–^mH_2_O, where M­(II) represents a divalent cation such as Co, Mg, Zn, Ni, or Cu, M­(III) is a trivalent cation such as Al, Cr, Fe, or Ga, A^n–^ is an anion with charge n, and m is the molar quantity of cointercalated water.

Iron-based hydrotalcite catalysts exhibit unique properties that make them advantageous for CO_2_ hydrogenation. These include high dispersion of active sites, tunable acidity and basicity,^18^ and the ability to incorporate various metal cations to modify catalytic behavior. Moreover, the structure of HTlc allows for a high degree of customization through multiple preparation routes (e.g., coprecipitation, urea hydrolysis, hydrothermal, sol–gel and microwave-assisted methods).^19^

Recent advancements in FT catalysts have focused on enhancing performance metrics such as activity, selectivity, and stability without adequately considering the environmental impacts of these catalysts throughout their entire lifecycle. Comprehensive Life Cycle Assessment (LCA) studies that evaluate the environmental impact of FT catalysts from production to disposal are notably lacking especially considering CO_2_ as feedstock for the process.^20^ This lack of catalyst-specific LCA studies limits our understanding of the sustainability of emerging FT catalysts and may lead to the uptake of options that are not environmentally robust in the long term. To address this gap, the present work combines a cradle-to-gate LCA study of hydrotalcite-derived catalyst synthesis with laboratory performance data for CO_2_ hydrogenation via the RWGS–FT route, enabling a direct link between catalyst choice, process performance and environmental impacts.

Life cycle assessment provides a holistic view by assessing the environmental impacts associated with all stages of a product’s life, from raw material extraction and catalyst manufacturing to operational use and end-of-life disposal. LCA has been increasingly applied to assess the environmental impacts of various experimental setups and processes in different scales, from laboratory to industrial processes, helping identify key areas where environmental impacts can be minimized. LCA results rely on the underlying inventory data describing the system (e.g., energy and material inputs, utilities, and waste management). Lab-scale LCAs based on primary inventory data can identify key material and energy hotspots.^21^ Firouzjaei et al. showed that electricity demand and chemical inputs can drive overall impacts in laboratory synthesis, and how chemical inputs and recycled feedstocks can help mitigate specific burdens.^22^ The LCA analysis is strongly dependent on the process performance, that is, on the capacity of the technology to transform the raw materials into the final products. In heterogeneous catalysis, this requires accounting for catalyst-dependent performance, since changes in catalyst formulation can affect conversion, selectivity, and overall yield, thereby modifying the inventory data (feed consumption, energy demand, and emissions per kg of product) and ultimately the LCA outcomes. Furthermore, operating the process requires energy, which entails additional CO_2_ emissions associated with electricity and utilities. Therefore, the environmental benefit of converting CO_2_ should be evaluated through a net CO_2_ balance, comparing the amount of CO_2_ converted in the reactor with the CO_2_ emitted across catalyst production and process operation.

Consequently, the scope of this paper is to conduct a detailed LCA of three different iron-based hydrotalcite catalysts tested at various temperatures, quantifying how catalyst choice and operating conditions affect the environmental impacts of FT and evaluating the associated CO_2_ balance. By integrating LCA with catalyst performance evaluations, this research aims to provide a comprehensive assessment of the environmental impacts and benefits associated with these catalysts. The goal is to identify not only the most efficient catalysts in terms of performance but also those that offer the lowest environmental footprint. Overall, the analysis supports the development of more sustainable FT pathways by guiding catalyst selection and process operation.

## 2 Methodology

In order to evaluate the environmental impact and the balance of the converted and produced CO_2_ from the FT reaction, the preparation methodologies and the conversion results obtained using three types of iron-based hydrotalcite catalysts will be analyzed. These catalysts were tested in a laboratory-scale plant with a continuous fixed-bed reactor, and the conversion and selectivity data to products were collected with different operating conditions of temperature and pressure.

### 2.1 Materials

Chemical reactants and catalysts precursors as Mg­(NO_3_)_2_^·^6H_2_O, Fe­(NO_3_)_3_^·^9H_2_O, Cu­(NO_3_)_2_^·^3H_2_O, Co­(NO_3_)_2_^·^6H_2_O, NaOH, NaHCO_3_ and KNO_3_ with analytical grade were purchased from Sigma-Aldrich. All reagents were used as received without further purification. Reacting gases for FT synthesis were used directly from high-purity (99.99%) cylinders purchased from Sapio Company.

### 2.2 Catalyst Synthesis

The catalysts identified in the study are MgCuFe30, MgCuFe40, and MgCoCuFe30. For simplicity, they are referred as Fe30, Fe40, and Co45. The three formulations were selected to probe (i) the effect of Fe content in hydrotalcite-derived catalysts (Fe30 vs Fe40) and (ii) the effect of partial Fe substitution with Co (Co45), which is known to influence activity and product selectivity in FT-related chemistry. All samples were synthesized by means of an ultrasound-assisted coprecipitation method and used as synthesized. High-power ultrasound irradiation was carried out by an Ultrasonic processors VC750 (Sonics and Materials) provided with a 13 mm diameter tip, operating at 750 W, 20 kHz, and 50% amplitude. Ultrasound enhances micromixing/cavitation during coprecipitation, minimizing local gradients and agglomeration, thus yielding smaller, more uniform LDH Substitute LDH with HTLc particles with improved compositional homogeneity and dispersion after activation. As a reference explanation, the synthesis of Fe30 is described hereafter: the synthesis was carried out by adding 50 mL of a 1 M NaOH and 2 M NaHCO_3_ solution dropwise to 56 mL of a 1 M solution of Mg, Fe, and Cu nitrate salts (molar ratio Mg/Fe/Cu = 13.2/6/1). During the addition process, the solution was sonicated with ultrasound for 3.5 min, and the sample was maintained at 5 °C. A yellow-brown solid immediately precipitated. At the end of the addition, 100 mL of distilled water was poured to suspend the precipitate. The solid was recovered by centrifugation and washed repeatedly with deionized water, then dried in an oven at 60 °C in air. The final composition of the catalysts obtained, determined by ICP-OES, are

$$\mathrm{“Fe30”}\left[{\mathrm{Mg}}_{0.67}{\mathrm{Cu}}_{0.045}{\mathrm{Fe}}_{0.29}{\left(\mathrm{OH}\right)}_{2}\right]{\left({\mathrm{CO}}_{3}\right)}_{0.15}0{.\mathrm{5 H}}_{2}O$$

$$\mathrm{“Fe40”}\left[{\mathrm{Mg}}_{0.55}{\mathrm{Cu}}_{0.05}{\mathrm{Fe}}_{0.4}{\left(\mathrm{OH}\right)}_{2}\right]{\left({\mathrm{CO}}_{3}\right)}_{0.20}0{.\mathrm{5 H}}_{2}O$$

$$\mathrm{“Co45”}\left[{\mathrm{Mg}}_{0.24}{\mathrm{Co}}_{0.42}{\mathrm{Cu}}_{0.05}{\mathrm{Fe}}_{0.29}{\left(\mathrm{OH}\right)}_{2}\right]{\left({\mathrm{CO}}_{3}\right)}_{0.15}0{.\mathrm{5 H}}_{2}O$$

### 2.3 Plant Setup and Reaction Conditions

During the CO_2_–FT reaction, which used H_2_ and CO_2_ as reactants, Brooks mass flow controllers were utilized to measure the flow rates of H_2_ (36 N mL/min, 99.99% purity), CO_2_ (12 N mL/min, 99.99% purity), and N_2_ (used as an inert internal standard, 5 N mL/min, 99.99% purity) within a continuous mixer. The detailed flow sheet is provided in a separate article.^17^ The experimental reactor had a 6 mm internal diameter and contained 1 g of a fresh catalyst. A blank test was conducted to confirm the inactivity of the plant’s inner surfaces, ensuring accurate results. The catalyst was positioned with a quartz wool bed divided into two sections. Heating was provided by a furnace, and the temperature was monitored using a K-type thermocouple, with an additional K-type thermocouple used to measure the reactor temperature. Heating and other lab utilities were supplied by mains electricity (Italian electricity grid). Catalyst activation was achieved by flowing a 2:1 molar ratio of H_2_/CO mixture (53 N mL/min) through the reactor for 4 h at 350 °C and 0.4 MPa. Liquid products, such as water and C7+ hydrocarbons (those with more than seven carbon atoms), were condensed at 5 °C in a 0.13 L cold trap with an external cooling jacket before being analyzed via gas chromatography. A pneumatic back pressure regulator maintained a pressure of 2.0 MPa. Permanent gases and noncondensable hydrocarbons were passed through another condenser and analyzed using an Agilent 3000A micro gas chromatograph to determine CO_2_ conversion (X_CO2_). This analysis was based on the peak areas of N_2_ and CO_2_ (A_N2_ and A_CO2_), their relative response factors (k), and the input flow rates of N_2_, and CO_2_ (F_in,N2_, and F_in,CO2_), as shown in eq 3

3 $$X_{CO2}=\frac{F_{\mathrm{in},CO2}-F_{\mathrm{out},CO_{2}}}{F_{\mathrm{in},CO2}}=1-\frac{F_{\mathrm{in},N2}}{F_{\mathrm{in},CO2}}\times k\frac{A_{CO2}}{A_{N_{2}}}$$

Oxygenated products were quantified from the condensed aqueous phase, assuming complete dissolution/collection in the water fraction; despite this approximation, the carbon balance closure error for all experiments remained below 5%. This liquid was analyzed using a Shimadzu 5000 A Total Organic Carbon instrument. For accurate measurement of gas compositions during the Fischer–Tropsch tests, a calibration procedure was employed using pure cylinders of CO_2_, CO, and H_2_. The flow rates of these gases were set by Brooks mass flow controllers and then quantified with a micro-GC analyzer, ensuring reliable baseline data for accurate quantification of reactants and products in the FT tests.

The FT tests with CO_2_/H_2_ mixtures were performed at three temperatures, 250, 300, and 350 °C using a molar ratio for the reactants CO_2_/H_2_/N_2_ molar ratio of 2:13:1. The furnace temperature was initially increased to 250 °C and held for about 24 h. Subsequently, it was raised to 300 °C and maintained for 24 h, and this process was repeated for the last temperature, 350 °C under 2 MPa of pressure.

### 2.4 LCA Methodology

Life Cycle Assessment was selected because laboratory performance metrics alone (e.g., conversion and selectivity) do not provide a complete picture of sustainability for CO_2_ hydrogenation processes and catalyst development. Catalyst production can be energy- and material-intensive, and process operation requires continuous utilities. Therefore, improvements observed at reactor level may be offset by upstream burdens, making it necessary to quantify trade-offs and prevent burden shifting between life-cycle stages. Based on this rationale, the scientific hypothesis tested here is that catalyst ranking based on reactor-level indicators may change once upstream catalyst manufacturing and operational utilities are included, potentially revealing net trade-offs across impact categories.

LCA provides a holistic framework to evaluate potential environmental burdens from raw material acquisition through manufacturing and use to end-of-life management, and it supports decision-making by identifying hotspots in resource use and emissions. In accordance with ISO 14040/14044, the assessment follows four interconnected phases: goal and scope definition (objectives, functional unit and system boundaries), life cycle inventory (LCI) compilation of all relevant inputs and outputs within the defined boundaries (materials, energy, utilities, emissions and waste), life cycle impact assessment (LCIA) to translate inventory flows into impact categories (e.g., climate change, eutrophication and resource depletion), and interpretation to identify key contributors and draw consistent conclusions.

In this study, these steps are applied iteratively by first defining the system boundaries and functional unit for the laboratory-scale FT tests, then compiling foreground inventories for catalyst preparation and FT operation together with background data for energy and material supply, calculating impacts with the selected LCIA methods, and finally comparing Co45, Fe30 and Fe40 while assessing the net CO_2_ balance (CO_2_ converted versus CO_2_ emitted to supply the required energy and materials) under different energy scenarios. To enable a consistent comparison among catalysts and operating conditions cradle-to-gate approach was used and the system was modeled as two coupled modules catalyst production and FT operation so that catalyst manufacturing burdens are propagated to impacts per 1 kg C_2_+ product.

In addition, scenario analysis of the electricity supply was performed to test how energy decarbonization affects the net CO_2_ balance and overall conclusions.

#### 2.4.1 System Boundaries

This LCA study is divided into two separate cradle-to-gate assessments, each with its own system boundary and functional unit. The first assessment focuses on the production of three different catalysts (Fe30, Fe40, Co45) for use in the Fischer–Tropsch process. The system boundaries encompass raw material extraction, including the gathering of necessary metals and chemical precursors, their transportation to the production site (modeled through SimaPro’s “market for” data, which accounts for average market processes including transportation), the chemical reactions and processes required to synthesize the catalysts. Catalyst end-of-life is not included in the study.

The second assessment focuses on the operational process of synthesizing C2+ hydrocarbons (i.e., all the hydrocarbons molecules with more than 1 carbon atom) using the Fischer–Tropsch method with the previously produced catalysts. This assessment includes catalyst use, energy consumption related to both reactor temperature maintenance and cold trap condenser unit, direct emissions from the chemical reaction, and the transportation of both raw materials and catalysts as accounted for by SimaPro’s “market for” data sets.

Both systems are depicted in the graph above Figure 1.

**Figure 1:**
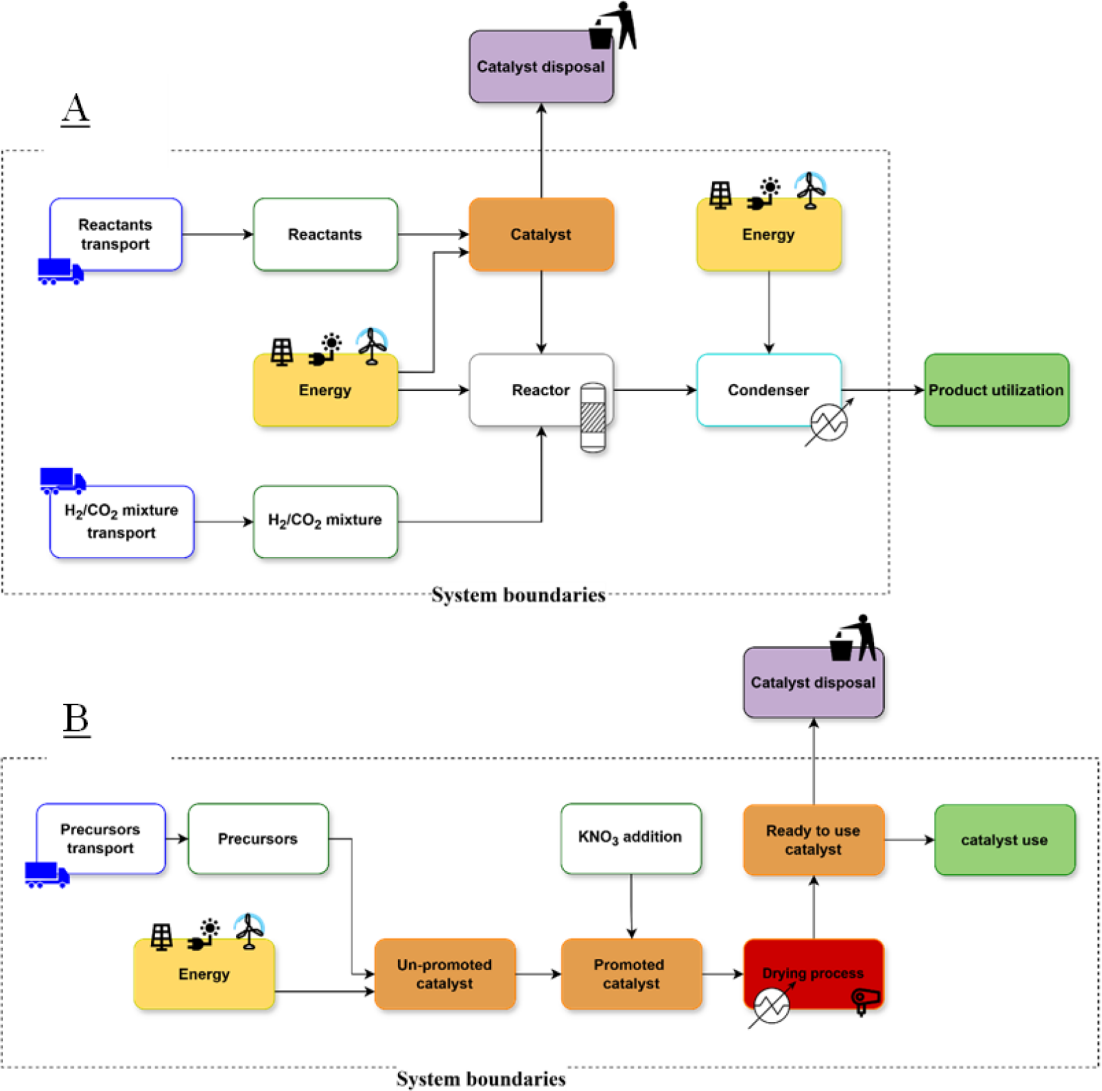
Schematic representation of the system boundaries for the Fischer–Tropsch process and catalyst production. (A) Process flow diagram for the Fischer–Tropsch synthesis, including reactant transport, reaction, product separation, and utilization. (B) Process flow diagram for catalyst preparation, highlighting precursor transport, promotion and drying.

#### 2.4.2 Functional Units

To adequately assess the outputs of the different phases, two distinct functional units were chosen. For the catalyst production phase, the functional unit is 1 kg of catalyst produced. For the Fischer–Tropsch operational phase, the functional unit is 1 kg of C2+ hydrocarbons produced. These units allow for a precise evaluation of the environmental impacts associated with each stage of the process.

#### 2.4.3 Inventory Data—Life Cycle Inventory

The life cycle inventory was built in SimaPro 9.4 software, by combining laboratory-scale information with background data sets. Laboratory data (measured/estimated) were used to model both catalyst synthesis and FT operation and included reagent inputs, utilities and energy demand for preparation steps, as well as operating utilities/energy demand and process outputs from the FT tests; these data were used to quantify the input/output flows associated with the defined functional unit. Upstream supply chains for materials and energy were modeled using the Ecoinvent database, selecting “market for” data sets to represent average supply mixes that include raw material extraction, production and transportation. Additional background processes were used to account for electricity generation and waste treatment where required, ensuring a consistent and comprehensive inventory for the assessed system.

#### 2.4.4 Life Cycle Impact Assessment

To assess the environmental impacts, three standardized methods were employed:

IPCC 2021 GWP100: this method was used to calculate the Global Warming Potential (GWP) in terms of CO_2_ equivalents, providing insight into the greenhouse gas emissions associated with both the Fischer–Tropsch process and the production of the catalysts.

CML-IA Baseline V3.08: a comprehensive method that evaluates a wide range of environmental impact categories, including abiotic depletion, ozone layer depletion, and various forms of ecotoxicity. It is particularly effective in identifying and quantifying the impacts associated with natural resource use and pollutant emissions.

Cumulative Energy Demand (CED) V1.11: this method was utilized to quantify the total energy demand of the system, distinguishing between renewable and nonrenewable energy sources. It plays a crucial role in assessing the energy efficiency of the catalyst production process and the Fischer–Tropsch operational phase, especially when comparing the sustainability of different energy sources.

## 3 Results and Discussion

### 3.1 Environmental Impact of Catalyst Production

The environmental impacts associated with the production of the three catalysts (Fe30, Fe40, Co45) are discussed based on the extrapolation of laboratory-scale data (5 g of catalyst) to an industrially relevant functional unit of 1 kg. While this approach allows for a direct calculation in the absence of industrial data, it does not fully capture the efficiency gains inherent to large-scale production. The results should therefore be interpreted as laboratory-scale estimates, while industrial-scale efficiencies are discussed qualitatively only.

Laboratory-scale processes typically exhibit lower material utilization efficiency, higher energy consumption, and increased waste generation per unit of product than industrial manufacturing, which can benefit from economies of scale, continuous operation, and process optimization. According to studies on scaling methodologies for chemical processes, direct linear extrapolation from small-scale experiments may overestimate impacts at larger scale.^23^

To refine environmental impact assessments, tools such as CatCost, developed by the National Renewable Energy Laboratory (NREL), provide standardized approaches for estimating catalyst manufacturing costs and environmental footprints while incorporating scale-up.^24^ ISO 14040/14044 standards emphasize the importance of defining functional units that reflect real-world industrial scenarios.^25^ Consequently, the environmental impact values reported here should be interpreted as a worst-case scenario, likely overestimating the real industrial burden. Future refinements may incorporate industrial LCA data sets and validated scale-up correlations to better approximate the environmental impacts of large-scale catalyst production.

#### 3.1.1 IPCC 2021 GWP100 Analysis

The Global Warming Potential (GWP) was measured using the IPCC 2021 GWP100 V1.01 method. This method evaluates the impact of greenhouse gases in terms of CO_2_ equivalents over a 100-year time horizon. The data collected indicate that the Co45 catalyst has a significantly higher impact on global warming compared to the Fe30 and Fe40 catalysts. Specifically, the CO_2_ equivalent emissions for all the three catalysts are reported in Table 1. The higher impact of Co45 sample is primarily attributable to the increased consumption of fossil energy during its production. The extraction and processing of cobalt, a key component of the Co45 catalyst, are energy-intensive processes that contribute significantly to its overall CO_2_ emissions.^26^ To better understand the sources of emissions contributing to GWP, Table 1 further breaks down the GWP100 into its fossil, biogenic, and land transformation components. The majority of emissions originate from fossil sources, with Co45 exhibiting the highest fossil GWP100 (60.53 kg CO_2_-eq). In particular, the Co45 catalyst shows higher emissions from biogenic sources (0.450 kg CO_2_-eq) and land transformation (0.13 kg CO_2_-eq) compared to the Fe30 and Fe40 catalysts. The biogenic CO_2_ emissions are associated with the use of biomass in the production process, while land transformation emissions are related to changes in land use for resource extraction and processing facilities. Although these contributions are smaller than those from fossil fuels, they still add to the overall GWP of the Co45 catalyst.

**Table 1:** Breakdown of Global Warming Potential (GWP100) for the Three Catalysts (Co45, Fe30, Fe40), Expressed in kg CO_2_-Equivalent (kg CO_2_-eq).^a^

Impact category	Unit	catalyst Co45	catalyst Fe30	catalyst Fe40
GWP100 - fossil	kg CO_2_-eq	60.53	44.90	44.95
GWP100 - biogenic	kg CO_2_-eq	0.450	0.365	0.36
GWP100 - land transformation	kg CO_2_-eq	0.13	0.028	0.03

a The values are categorized into fossil, biogenic, and land transformation emissions.

The Fe30 and Fe40 catalysts exhibit lower GWP values, suggesting a more sustainable production process in terms of greenhouse gas emissions. This is largely due to less energy-intensive raw material extraction and processing, as well as more efficient energy use. Fe30 and Fe40 show very similar impact values across most categories. This is consistent with their comparable catalyst formulation and preparation route, which result in closely aligned precursor requirements and drying/washing utilities per kg of catalyst. Consequently, differences in Fe loading translate into only marginal changes in the life-cycle inventory and associated impacts. A similar trend is observed for Fe30 and Fe40 in the other impact assessment methods applied in this study (Sections 3.1.2 and 3.1.3). Therefore, the selection of the catalyst has a significant impact on the overall carbon footprint of the Fischer–Tropsch process.

#### 3.1.2 Cumulative Energy Demand (CED) Analysis

The Cumulative Energy Demand (CED) analysis, shown in Figure 2, provides a comprehensive view of the total energy required during the life cycle of the catalysts, including both renewable and nonrenewable energy sources. This analysis is crucial for understanding the overall energy efficiency and sustainability of the production processes for Co45, Fe30, and Fe40 catalysts.

**Figure 2:**
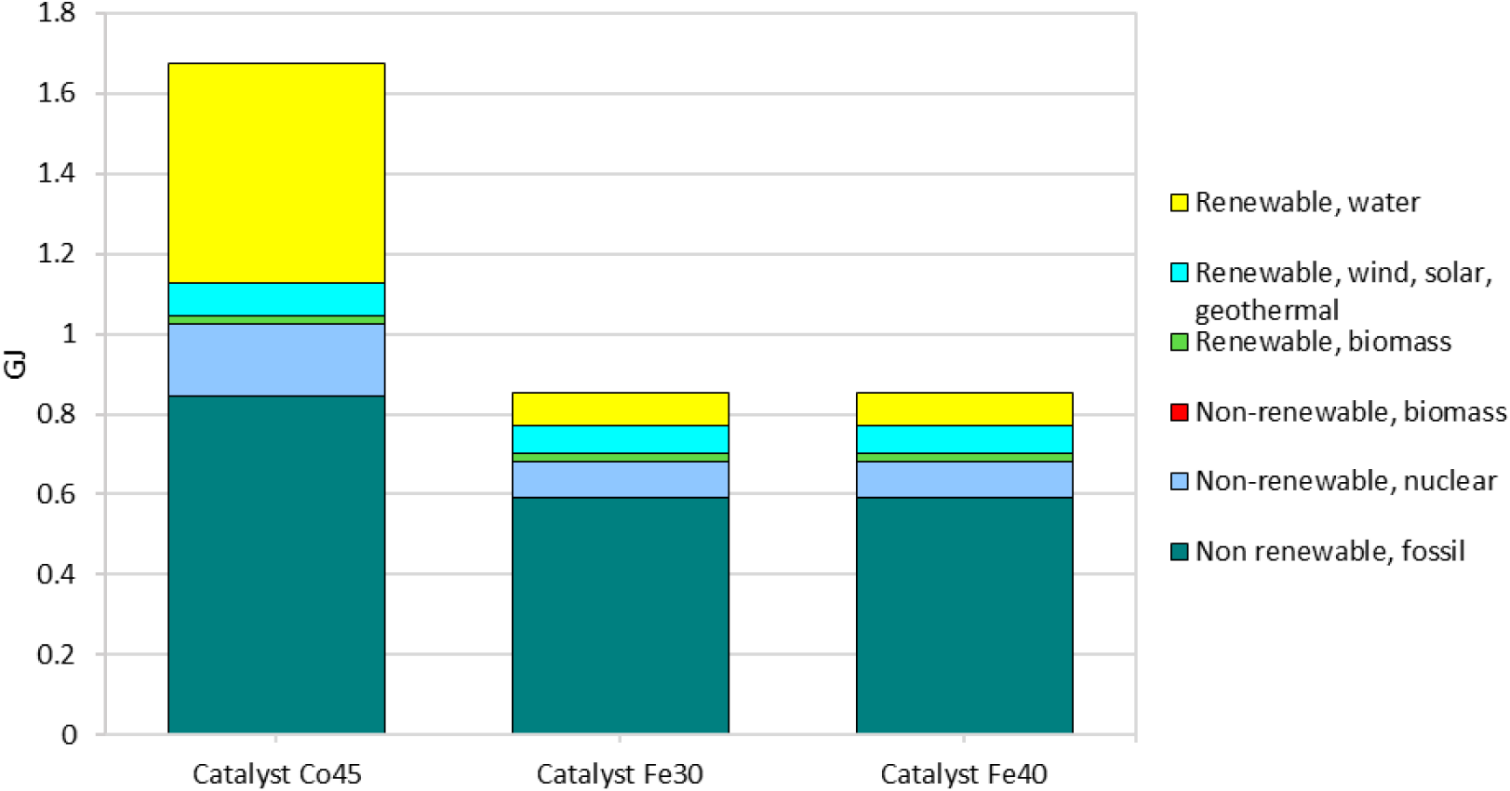
Energy consumption for the production of catalysts Co45, Fe30, and Fe40, categorized by energy source (renewable and nonrenewable).

The total energy demand for producing 1 kg of each catalyst reveals significant differences. The Co45 catalyst requires nearly double the total energy compared to Fe30 and Fe40, with Co45 consuming 1.676 GJ of energy versus 0.853 GJ for both Fe30 and Fe40. This substantial difference highlights the significantly higher energy input needed for Co45 production.

The Co45 catalyst exhibits a notably higher demand for nonrenewable energy, mainly fossil fuels and, to a lesser extent, nuclear energy. This is a critical factor contributing to its higher Global Warming Potential and overall environmental impact.

Nuclear energy consumption for Co45 production is also notably higher. While nuclear energy has a lower carbon footprint compared to fossil fuels, it poses other environmental challenges, including the management of radioactive waste. The higher use of nuclear energy in Co45 production reflects the complex energy requirements of processing cobalt.

In terms of renewable energy, Co45 also demands more from sources such as biomass, wind, solar and hydroelectric power. The use of hydroelectric power is particularly high, suggesting that while there is an effort to incorporate cleaner energy sources, the overall energy demand remains high. The renewable energy inputs, although higher for Co45, do not sufficiently offset the overall greater energy requirements compared to the iron-based catalysts.

The iron-based catalysts, Fe30 and Fe40, show a more balanced energy profile, with lower total energy consumption and a more efficient use of both nonrenewable and renewable energy sources. Their production processes are less energy-intensive, leading to lower overall environmental impacts. This balance is reflected in their nearly identical CED profiles, indicating minimal differences in their composition and production processes.

#### 3.1.3 CML-IA Baseline Analysis

The environmental impacts associated with the production of the three catalysts were assessed using the CML-IA baseline V3.08 method, shown in Figures 3 and 4. This method provides a comprehensive evaluation across various impact categories, including abiotic depletion, global warming potential, ozone layer depletion, human toxicity, ecotoxicity (freshwater, marine, and terrestrial), photochemical oxidation, acidification, and eutrophication.

**Figure 3:**
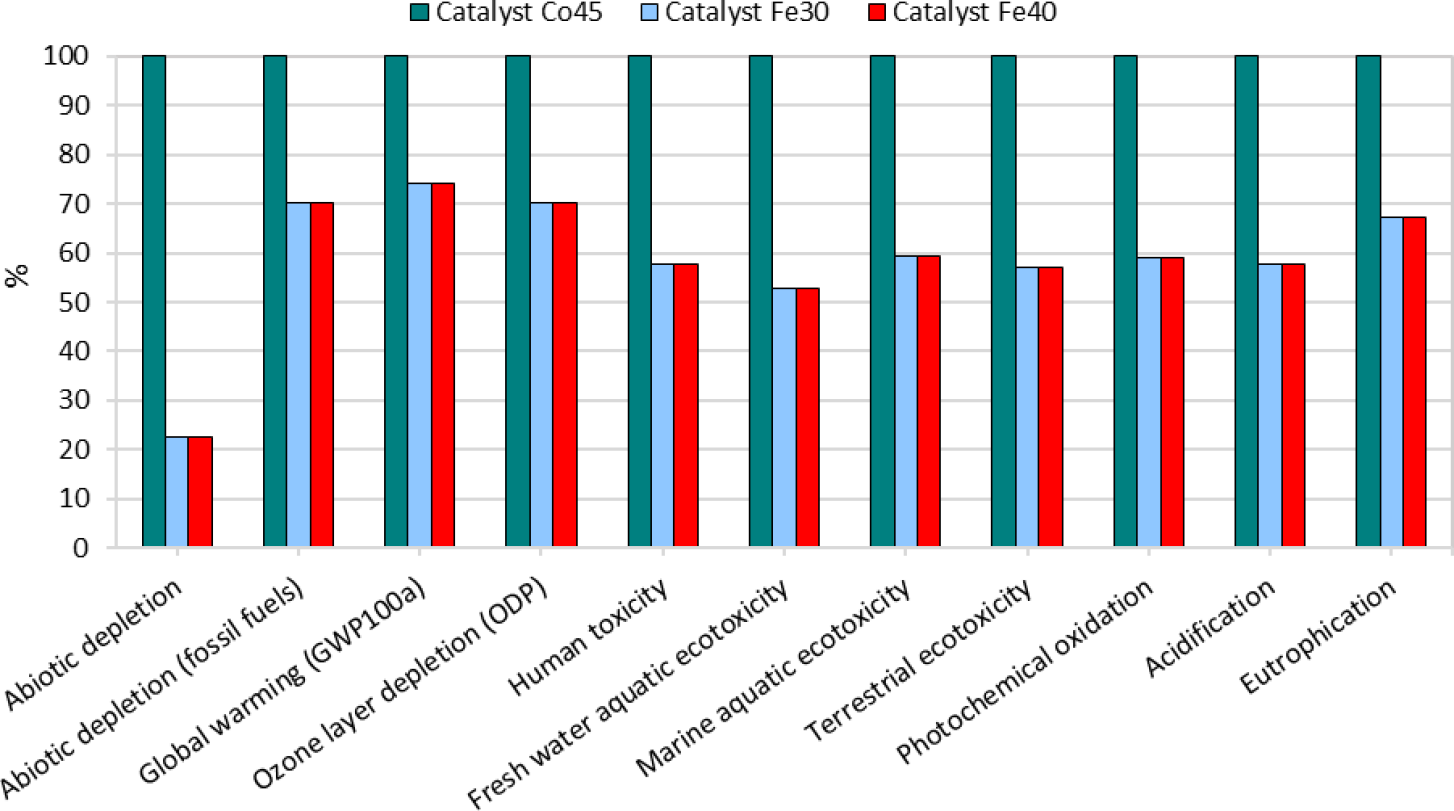
Comparative environmental impact assessment across multiple impact categories, expressed as a percentage of the highest value in each category (Characterization).

**Figure 4:**
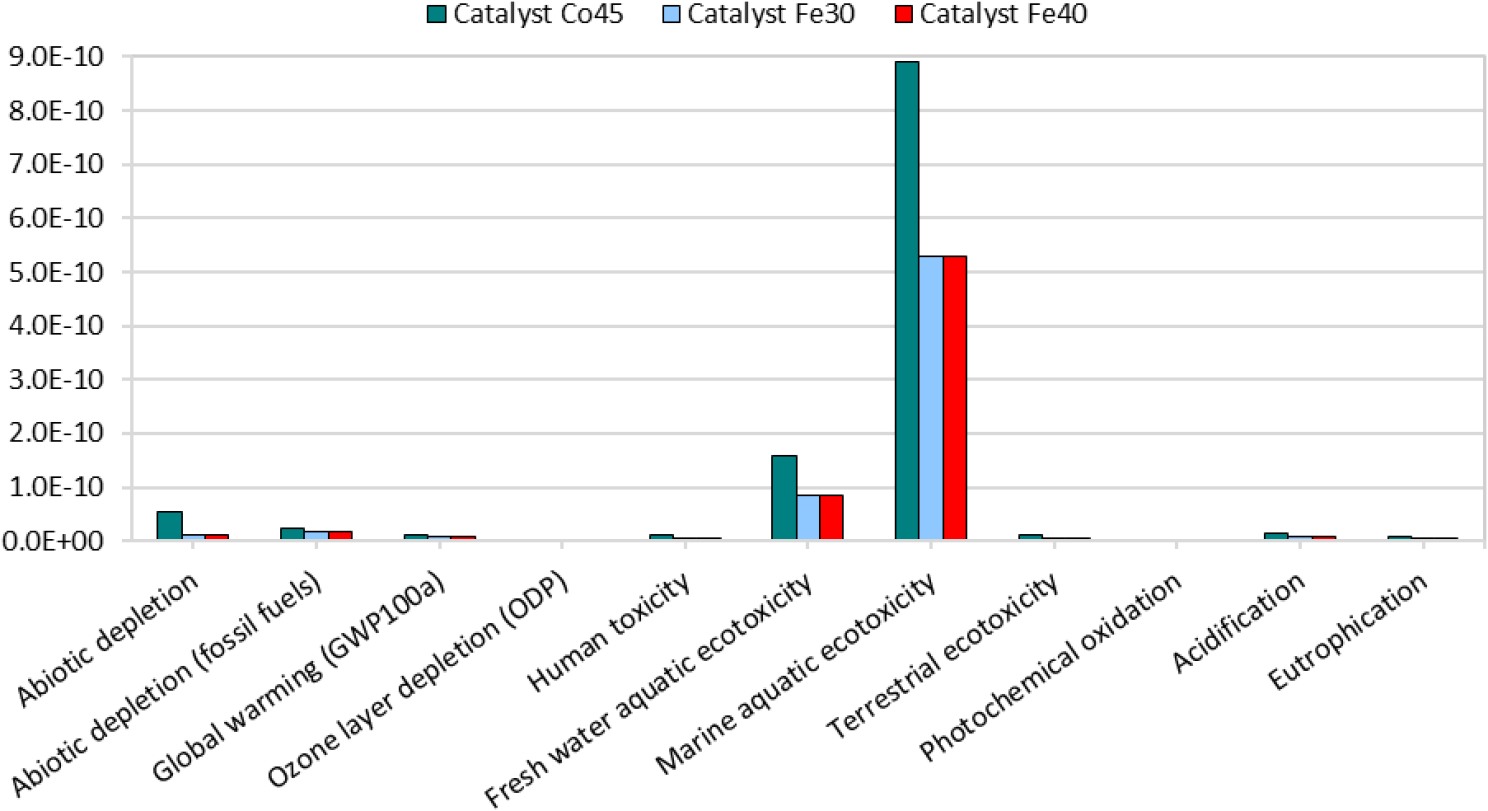
Environmental impact assessment across multiple categories, expressed in absolute values (normalization).

Abiotic depletion measures the consumption of nonliving resources. Co45 exhibited a significantly higher impact in this category compared to Fe30 and Fe40, primarily due to the extensive use of cobalt. This catalyst’s production demands a substantial amount of mineral resources, reflecting a notable environmental burden.

The Global Warming Potential of Co45 was also the highest among the catalysts, as discussed in Section 3.1.1. Ozone layer depletion impacts were higher for Co45, linked to the emissions of ozone-depleting substances associated with the upstream supply chain. Human toxicity was notably higher for Co45 as well, attributed to the release of hazardous substances during its production, which poses significant health risks.^27^

In terms of ecotoxicity too, Co45 demonstrated the highest impacts across freshwater, marine, and terrestrial categories. The production of this catalyst involves significant releases of pollutants, particularly cobalt sulfate, which contribute to its elevated ecotoxicity scores. The marine aquatic ecotoxicity, in particular, was markedly higher due to waste disposal issues associated with cobalt processing.

Photochemical oxidation, associated with smog formation, was also highest for Co45. This was primarily due to the emission of volatile organic compounds (VOCs) along its upstream supply chain. Acidification potential, which can lead to acid rain, was another category where Co45 had a significantly higher impact, mainly due to sulfur dioxide and nitrogen oxide emissions. Lastly, eutrophication, indicating nutrient enrichment of water bodies, was higher for Co45, reflecting the environmental challenges posed by nutrient runoff from cobalt mining activities.

Overall, Figure 3 shows that Co45 is the highest-impact catalyst in all 11 CML categories, whereas Fe30 and Fe40 remain consistently lower and closely aligned. The largest relative difference between Co45 and the Fe-based catalysts is observed for abiotic depletion, while the gap is smaller for global warming (GWP100a).

Normalization, shown in Figure 4, is essential for contextualizing results, enabling a comparison of impact categories based on their relative significance rather than their absolute values.

In the analysis of the catalysts Co45, Fe30, and Fe40, marine aquatic ecotoxicity is identified as the most significant impact category, indicating a considerable risk to marine ecosystems. This is followed by freshwater aquatic ecotoxicity, which also demonstrate substantial, though comparatively lower, impacts. These findings underscore the primary environmental concerns associated with these catalysts, particularly their potential contribution to ecological toxicity in aquatic environments. Conversely, categories such as ozone layer depletion and photochemical oxidation exhibit minimal impacts, suggesting they are of lesser concern for these specific catalysts.

Consequently, normalization plays a critical role in prioritizing environmental impacts, directing attention to the most severe risks.

### 3.2 Environmental Impact of FT Product Synthesis

#### 3.2.1 Catalytic Results

The catalytic performance in FT synthesis of the three catalysts (Fe30, Fe40, and Co45) was evaluated in terms of CO_2_ conversion and product selectivity across a range of temperatures (250 to 350 °C). The results provide valuable insights into the reaction mechanisms and the influence of catalyst composition on the distribution of products.

The CO_2_ conversion rates increase with temperature (Figure 5) for all three catalysts, as expected, due to the enhanced kinetic energy that facilitates reaction rates. However, Co45 exhibits a notably higher conversion efficiency compared to the Fe-based catalysts across the entire temperature range. At 350 °C, Co45 reaches a CO_2_ conversion rate of approximately 50%, significantly outperforming Fe30 and Fe40, which plateau at around 35% and 30%, respectively. The superior performance of Co45 can be attributed to the inherent properties of cobalt, as supported by experimental evidence showing that cobalt oxide phases can be highly active in Fischer–Tropsch synthesis and CO_2_ hydrogenation, with activity influenced by oxidation state and support effects.^28^ Moreover, Co-based catalysts are relevant for higher-carbon products due to their ability to promote chain growth.^29^ Efficient H_2_ activation on cobalt catalysts can enhance hydrogenation kinetics and contribute to higher CO_2_ conversion.^30^ In addition, mechanistic studies highlight that CO_2_ activation and the dominant reaction pathway depend on cobalt oxidation state, which influences overall conversion.^31^ The Fe-based catalysts, while still effective, show a more gradual increase in conversion, reflecting the slower reaction kinetics associated with iron.

**Figure 5:**
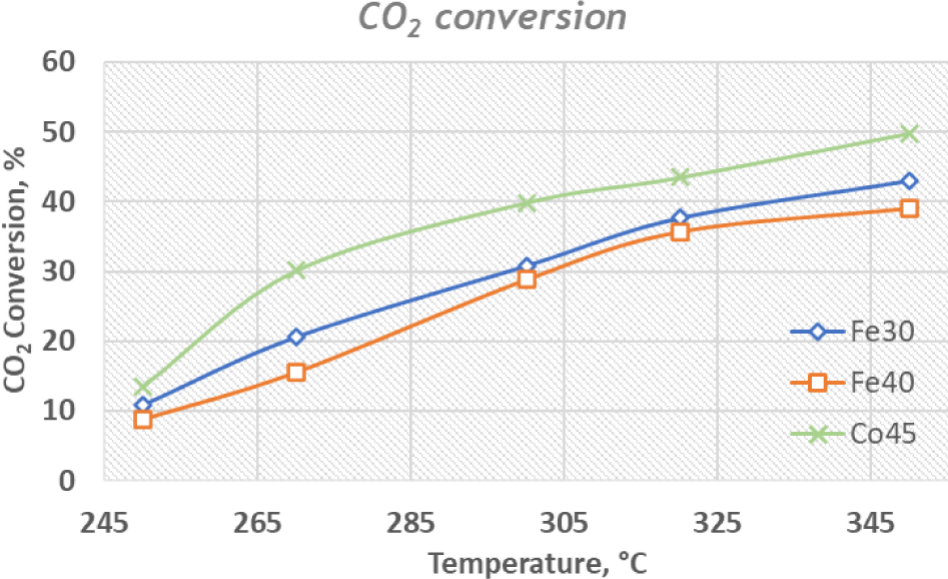
CO_2_ conversion as a function of temperature for catalysts Fe30, Fe40, and Co45.

The selectivity of products CH_4_, CO, light hydrocarbons (C_2–6_), and heavier hydrocarbons (C_7+_) reveals critical differences in the catalytic behavior of the three catalysts (Figure 6). It is important to note that C_2_+ selectivity refers to the sum of light hydrocarbons (C_2_-_6_) and heavier hydrocarbons (C_7_+).

**Figure 6:**
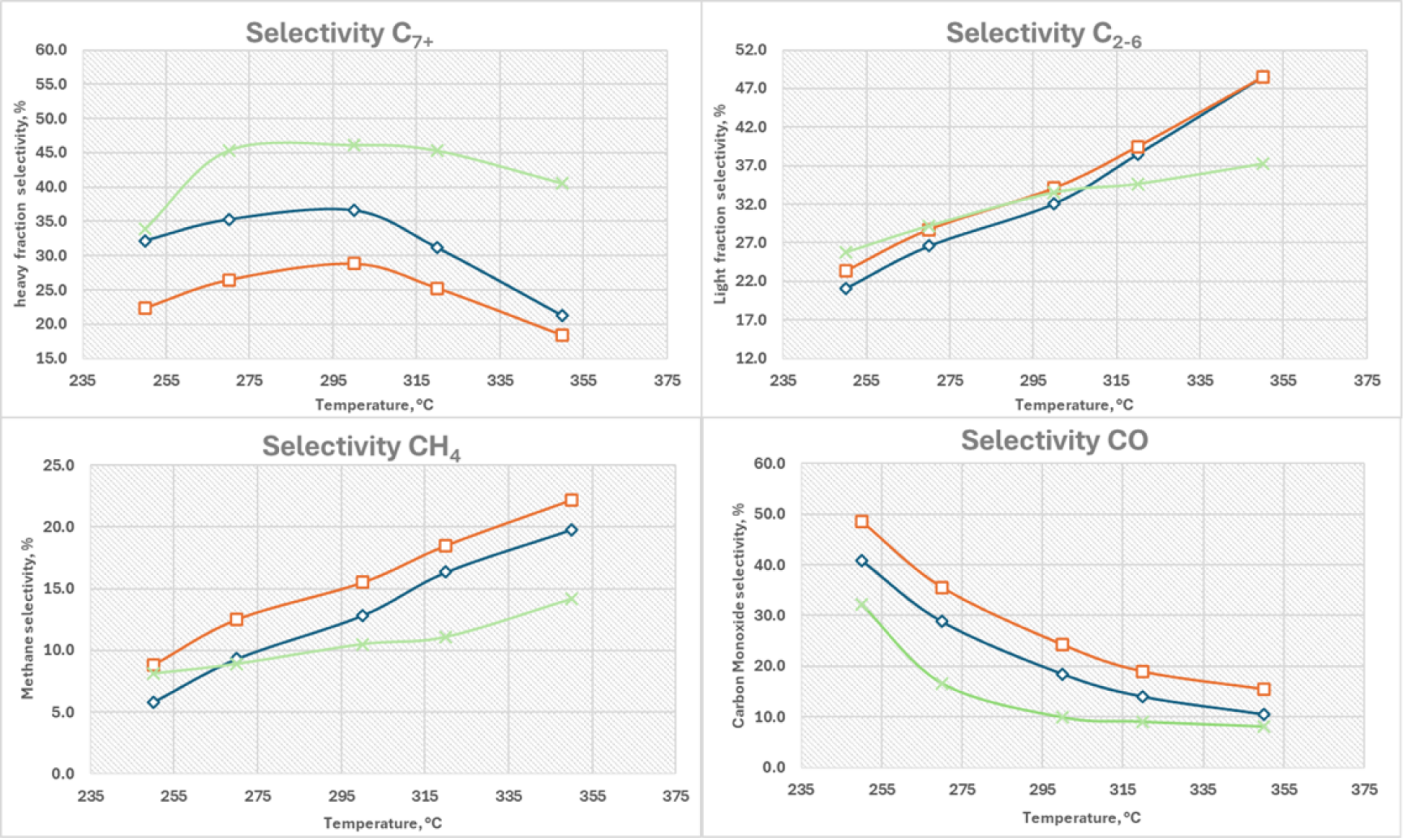
Selectivity trends of different products as a function of temperature. The top-left plot shows C_7_+ (heavy fraction) selectivity, the top-right plot presents C_2_–_6_ (light fraction) selectivity, the bottom-left plot displays CH_4_ selectivity, and the bottom-right plot illustrates CO selectivity. The blue curve corresponds to the Fe30 catalyst, the orange curve to Fe40, and the green curve to Co45.

Regarding methane selectivity, it increases with temperature for all catalysts, but the Fe40 catalyst consistently shows the highest selectivity for CH_4_, reaching nearly 22% at 350 °C. This could be due to Fe40s higher iron content, which favors the production of lighter hydrocarbons like methane.^32,33^ Cobalt-based Co45, while less selective for CH_4_, shows a moderate increase, indicating a balance between methane production and the formation of longer-chain hydrocarbons. On the contrary, CO selectivity decreases as temperature rises, which aligns with the expected behavior of Fischer–Tropsch reactions where higher temperatures drive further hydrogenation of CO to hydrocarbons. Fe40 exhibits the highest CO selectivity at lower temperatures, suggesting that it is less effective at progressing the reaction toward complete hydrocarbon formation. Co45, in contrast, shows the lowest CO selectivity, reinforcing its superior catalytic efficiency in converting CO_2_ to hydrocarbons rather than intermediate products like CO.

Shifting attention to the hydrocarbon phases: the selectivity for light hydrocarbons (C_2–6_) increases with temperature for all catalysts. Fe40 again shows the highest selectivity in this category, particularly at temperatures above 325 °C. This trend suggests that Fe40 favors the formation of shorter-chain hydrocarbons, likely due to its iron composition, which promotes chain termination reactions leading to lighter hydrocarbon fractions whereas heavy hydrocarbon selectivity (C_7+_) decreases with temperature for Fe40, but interestingly, Co45 exhibits a peak in heavy hydrocarbon selectivity at around 305 °C, after which it slightly declines. This peak suggests that Co45 is particularly effective at producing longer-chain hydrocarbons under moderate temperature conditions, making it an attractive catalyst for applications where the production of heavier hydrocarbons is desired.

The differing behaviors of Fe30, Fe40, and Co45 can be explained by the distinct catalytic properties of iron and cobalt. Iron catalysts, particularly Fe40 with higher iron content, tend to produce lighter hydrocarbons and demonstrate higher CO and CH_4_ selectivity. This could be due to the fact that iron catalysts facilitate the Boudouard reaction (2CO → CO_2_ + C) and water–gas shift (CO + H_2_O → CO_2_ + H_2_) reactions, which can compete with the Fischer–Tropsch process, leading to higher methane and CO production.^31^

Cobalt catalysts like Co45, on the other hand, are more efficient in hydrogenating CO_2_ to longer-chain hydrocarbons, as evidenced by their lower CO selectivity and higher C_7+_ selectivity. Cobalt’s ability to maintain a higher hydrogenation activity, even at elevated temperatures, allows for the production of heavier hydrocarbons, which are desirable in many industrial applications.

#### 3.2.2 LCA Results

On the basis of the experimental results presented in the previous section, we evaluated the environmental impacts associated with three catalysts: Co45, Fe30, and Fe40, each operating at their optimal temperature of 350 °C. The functional unit in this study is defined as 1 kg of product obtained. Here, the product refers to C_2_+ hydrocarbons, ensuring a consistent and comparable assessment of environmental impacts across different impact categories. The evaluation is conducted using the methodologies described in Chapter 2.4.3 Inventory Data.

##### 3.2.2.1 CML-IA Baseline Analysis

The CML-IA baseline method evaluates a broad spectrum of environmental impact categories. From the data, it is evident that the Fe40 catalyst exhibits the highest impact across nearly all categories, a trend that is strongly correlated with its lower catalytic performance compared to the other two catalysts. This phenomenon is consistent with previous discussions where the catalytic efficiency directly influences the environmental burden; higher efficiency typically translates to lower environmental impact per unit of product. Overall, Figure 7 shows that Fe40 exhibits the highest impacts across all 11 CML categories for C_2_+ production at 350 °C. In most impact categories, Fe30 displays intermediate values, whereas Co45 shows the lowest relative impacts. An exception is observed for abiotic depletion, where Co45 exceeds Fe30, indicating a comparatively higher contribution of Co45 to resource depletion despite its lower impacts in the other categories.

**Figure 7:**
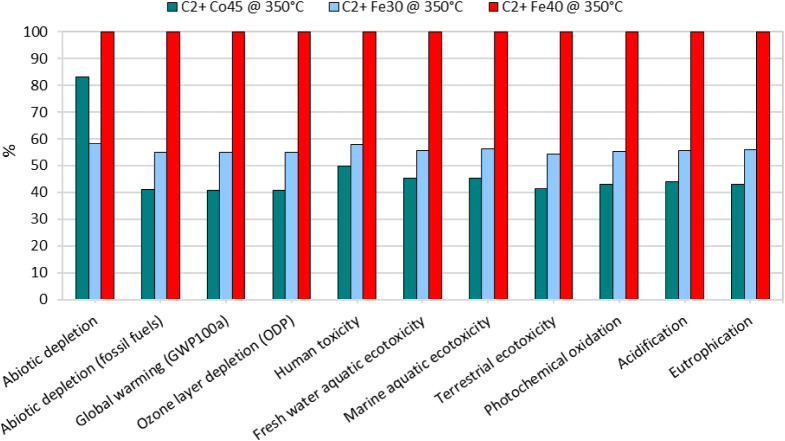
Comparative environmental impact assessment of C_2_+ hydrocarbon production at 350 °C for catalysts Co45, Fe30, and Fe40 across multiple impact categories.

This significant difference can be attributed to the greater energy consumption required for Fe40s operation and its lower efficiency in converting CO_2_ into desired products. The Co45 catalyst, despite its higher initial environmental impact during production, shows a markedly lower impact in the operational phase due to its superior catalytic performance. Notably, in categories such as photochemical oxidation, terrestrial ecotoxicity, and eutrophication, the Fe40 catalyst again shows higher impacts, underscoring its inefficiency. The increased impact in terrestrial ecotoxicity and eutrophication for Fe40 is likely related to the byproducts and emissions generated during its operation, including the treatment of wood ash mixture and power sawing for wood chips, which are used in the energy mix.

Figure 8 indicates that marine aquatic ecotoxicity dominates the overall impact profile for C_2_+ production at 350 °C, while the remaining categories contribute only marginally.

**Figure 8:**
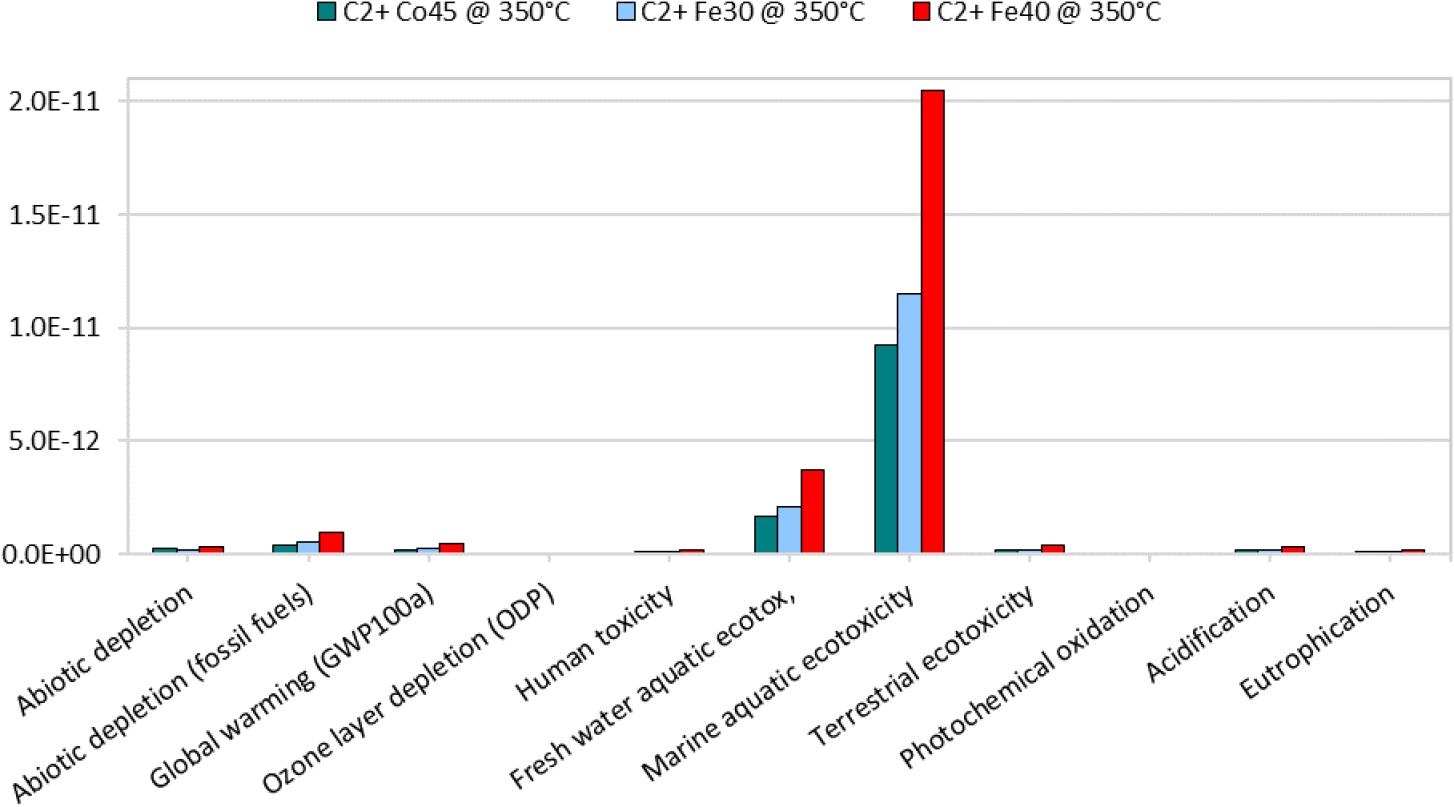
Absolute environmental impact values for C_2_+ hydrocarbon production at 350 °C (normalization).

##### 3.2.2.2 IPCC 2021 GWP100 Analysis

The Global Warming Potential (GWP) analysis using the IPCC 2021 GWP100 method reveals that Fe40 also has the highest impact in terms of CO_2_-eq emissions, registering 2.39 kg CO_2_-eq per kg of product. This is significantly higher than Fe30 (1.31 kg CO_2_-eq) and Co45 (0.976 kg CO_2_-eq), Figure 9.

**Figure 9:**
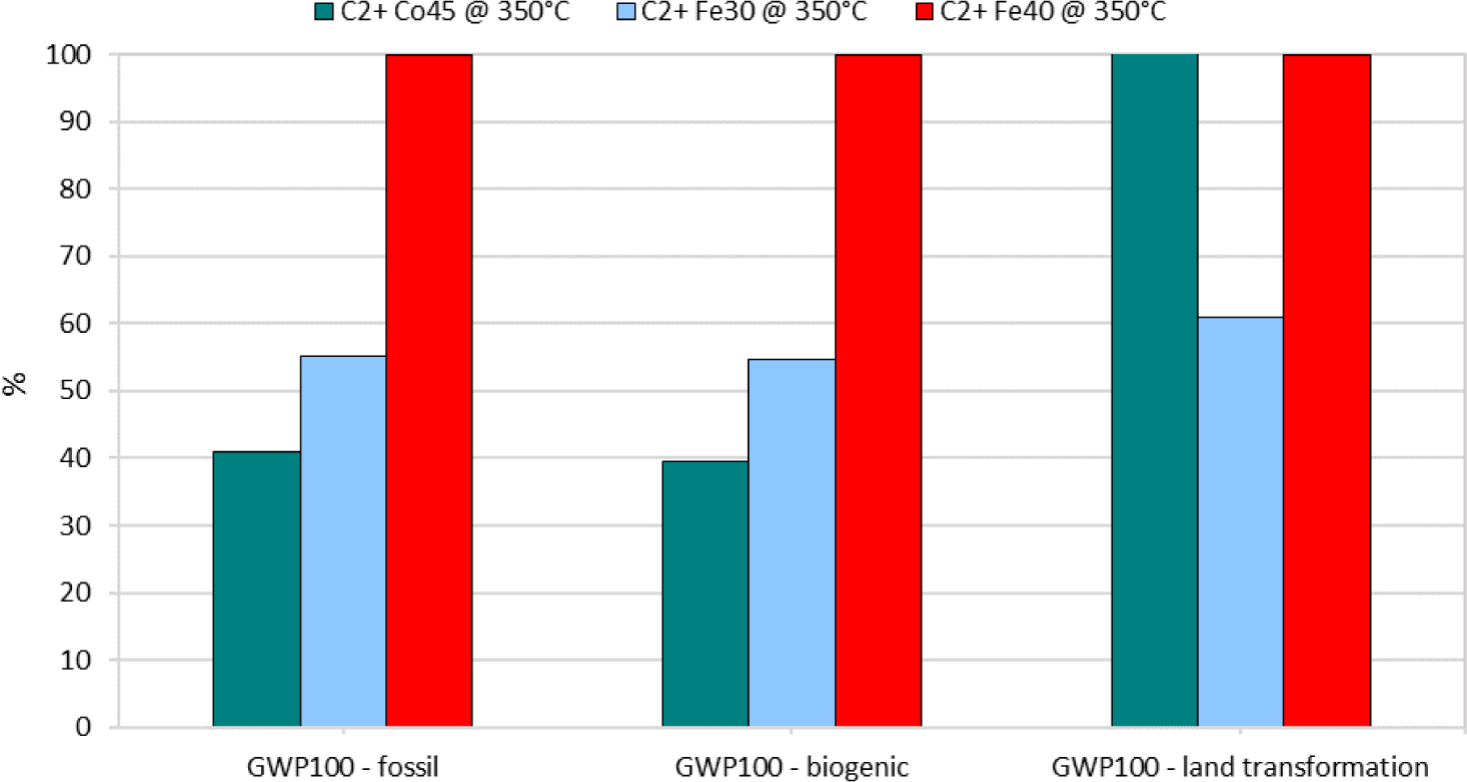
Breakdown of Global Warming Potential (GWP100) contributions for C_2_+ hydrocarbon production at 350 °C using catalysts Co45, Fe30, and Fe40.

The GWP100 analysis is segmented into fossil, biogenic, and land transformation categories. Fe40 exhibits the highest impact in the fossil and biogenic categories, indicating its reliance on nonrenewable energy sources and the higher carbon footprint associated with its operation. Interestingly, in the land transformation category, the Co45 catalyst shows a slightly higher impact than Fe40, which is primarily due to the specific characteristics of cobalt sulfate used in the catalyst, particularly the electricity consumption linked to the cobalt industry.

##### 3.2.2.3 Cumulative Energy Demand (CED) Analysis

The CED analysis further supports the findings of the CML and GWP analyses, highlighting critical differences in the energy demands of the three catalysts for the Fischer–Tropsch synthesis of C2+ hydrocarbons. Co45 shows the lowest total energy consumption at 22.227 MJ/kg of product, primarily due to its lower reliance on nonrenewable fossil energy (13.761 MJ).

In comparison, Fe30 and Fe40 require 28.121 MJ and 51.382 MJ, respectively, underscoring Co45’s superior efficiency (Figure 10). The reduced energy demand of Co45 is particularly significant, with a 40.94% lower fossil energy use compared to Fe40. Furthermore, Co45 also performs better in the utilization of renewable energy sources, especially in renewable water energy (3.576 MJ), suggesting a more balanced and resilient energy profile. This balanced use of renewable and nonrenewable resources further supports the sustainability of Co45, making it less vulnerable to energy supply fluctuations. The nuclear energy demand for Co45 is lower than for Fe30 and Fe40, at 2.448 MJ, enhancing its versatility and sustainability.

**Figure 10:**
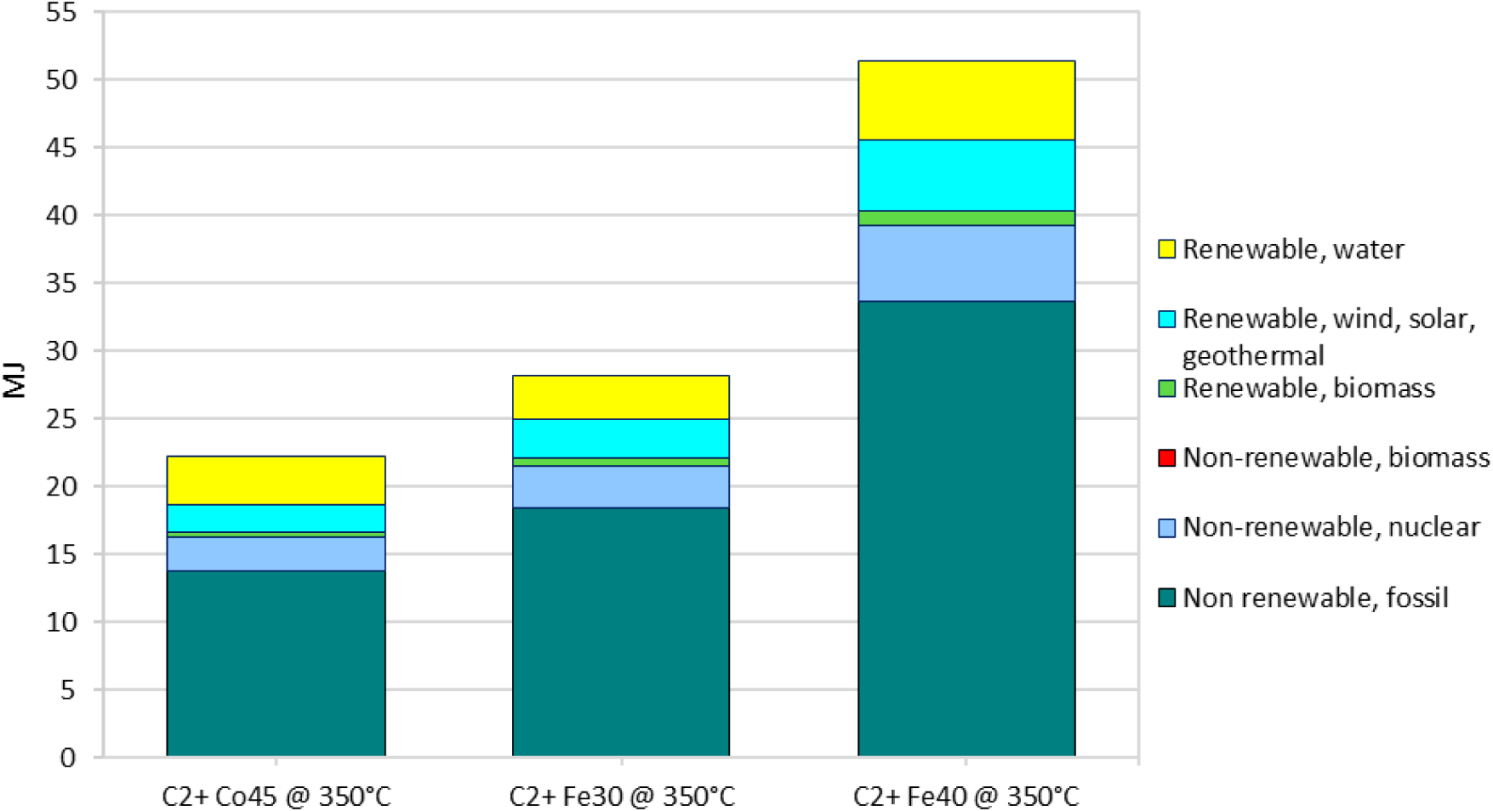
Energy consumption breakdown for C_2_+ hydrocarbon production at 350 °C.

### 3.3 Carbon Neutrality Evaluations—Three Catalysts in Comparison

Carbon neutrality aims to balance emitted and converted CO_2_, making it a key target for sustainable industrial processes. In chemical and fuel production, reducing carbon footprints is particularly challenging, but CO_2_ hydrogenation offers a promising path.^34^ However, achieving true carbon neutrality depends on coupling CO_2_ conversion with low-carbon energy inputs, since carbon-neutral pathways combine CO_2_ reduction with clean energy technologies.^35^ It also depends on upstream feedstock and energy choices, including routes that valorize waste/biomass into biofuels and their associated process requirements.^36^ More broadly, the “green carbon science” perspective frames carbon neutrality as optimizing carbon resource processing, utilization, CO_2_ fixation and recycling to minimize net CO_2_ emissions.^37^ This study evaluates the carbon neutrality potential of FT by comparing Co45, Fe30, and Fe40 catalysts under different energy scenarios. The assessment of carbon neutrality across the three catalysts Co45, Fe30, and Fe40 requires an analysis of their CO_2_ Utilization Factor (CUF), a metric representing the proportion of CO_2_ converted relative to the CO_2_ produced in the Fischer–Tropsch synthesis process.

4 $$CO_{2}\;\mathrm{utilization factor},\%=\frac{kg\;of\;CO_{2}\;\mathrm{converted}}{kg\;of\;CO_{2}\;\mathrm{produced}}\times 100$$

where CO_2_ converted refers to the amount of CO_2_ transformed from raw material to products, while CO_2_ produced represents the amount of CO_2_ emitted in order to generate the energy needed for the process. This indicator is critical in evaluating the net carbon balance, where values above 100 indicate a net CO_2_-consuming process, then potentially contributing to carbon-negative fuels. For example, a CUF of 200% means that for 1 mol of CO_2_ produced, 2 mol of CO_2_ are simultaneously converted into products. This implies that the process is offsetting its own emissions and also actively removing additional CO_2_ from the system. In contrast, a CUF of 50% signifies that for every mole of CO_2_ produced, only 0.5 mol of CO_2_ are converted, meaning that more CO_2_ is being generated than utilized. This results in a net CO_2_-emitting process. The CUF values at different reaction temperatures are summarized in Table 2. At 250 °C, all catalysts exhibit low conversion, with Fe40 achieving the lowest CUF, indicating only 13% of the CO_2_ produced is effectively converted, while Co45 achieves 16% utilization. However, it is important to note that these CUF values are derived from laboratory-scale experiments, where gas flow rates, reaction residence times, and thermal management are not optimized to the extent possible in an industrial-scale system. In an industrial plant, process parameters are fine-tuned to maximize CO_2_ conversion efficiency, leading to higher CUF values than those reported in this study. Thus, the numbers presented here should be interpreted as a conservative estimate rather than absolute values representative of large-scale operations. As the temperature increases to 300 °C, the CUF improves significantly, particularly for Co45 and Fe30, reaching 74% and 90%, respectively, suggesting enhanced CO_2_ utilization at elevated temperatures. The highest CUF values are observed at 350 °C, where Co45 achieves 167%, indicating that it converts 167% of the CO_2_ that it produces, making it net carbon negative. Similarly, Fe30 reaches 130 CUF change in 130 CUF %, reinforcing its high catalytic efficiency. The increase in CUF with temperature can be attributed to the enhanced kinetics of CO_2_ hydrogenation, where higher temperatures improve reaction rates and increase the selectivity toward longer-chain hydrocarbons, effectively capturing more CO_2_ into liquid fuel intermediates.^38^ However, this improved CO_2_ utilization comes with trade-offs, particularly in energy demand and environmental burden. While Co45 and Fe30 demonstrate high CUF at optimal temperatures, their production phase indicates substantial impacts in abiotic depletion and fossil fuel consumption, requiring a broader perspective when evaluating sustainability.^39^ An essential consideration is the interaction between energy source and CUF. While a higher CUF generally correlates with improved sustainability, the energy mix used to drive the synthesis can offset or enhance these benefits. For instance, using hydroelectric power significantly reduces GWP, reinforcing the benefits of catalysts with high CUF. In contrast, fossil fuel-based energy sources may counteract the net CO_2_ capture benefits, diminishing the catalysts’ ability to achieve carbon neutrality.

**Table 2:**
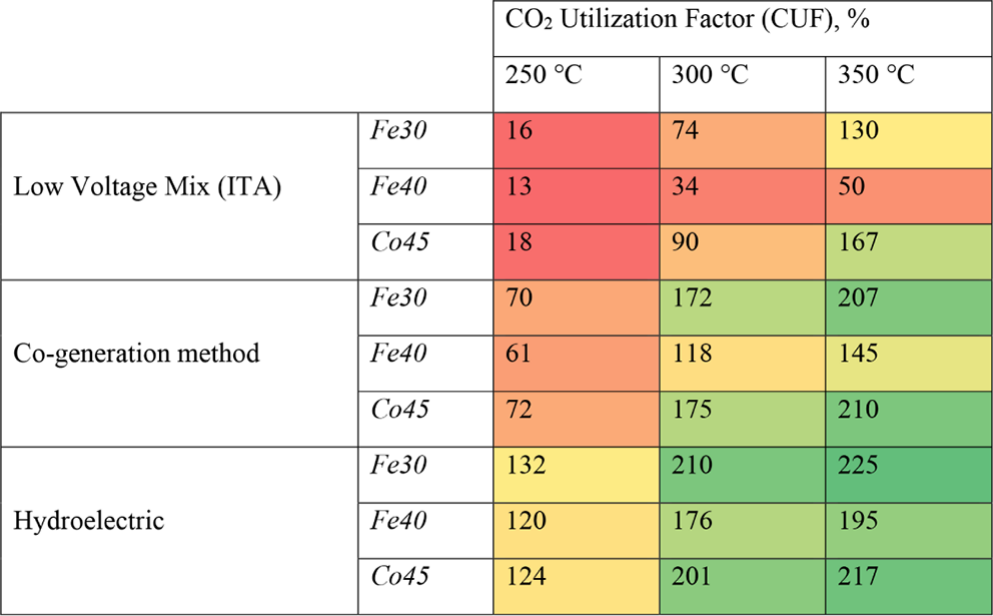
Comparison between the Catalysts Regarding CO_2_ Utilization Factor Considering Different Energy Sources for the System and Temperatures.^a^

a The color gradient represents the magnitude of the CO_2_ utilization factor (CUF), with red indicating lower values, yellow representing intermediate values, and green corresponding to the highest CUF values.

The findings indicate that catalysts alone, tested in a laboratory-scale plant, cannot achieve full carbon neutrality without integrating low-carbon energy sources. While Fe30 achieves the highest CUF at 350 °C, the Co45 catalyst presents a more balanced profile by maintaining a high CUF with lower energy demand compared to Fe30. However, it is important to note that at the laboratory-scale, energy consumption per unit of product is significantly higher than at the industrial scale, which may influence the relative impact of different catalyst-energy combinations. The selection of the optimal catalyst-energy combination is, therefore, essential for minimizing the net environmental impact of the Fischer–Tropsch process.^40^ The choice of energy source in Fischer–Tropsch synthesis is a fundamental determinant of whether the process can achieve net CO_2_ reduction or even become carbon-negative.^41^ While catalyst selection plays a key role in CO_2_ conversion efficiency, the environmental impact is significantly shaped by the carbon intensity of the energy input. The transition from fossil-based grid electricity to renewable sources such as hydroelectric power or biomass cogeneration introduces substantial improvements in CO_2_ Utilization Factor (CUF) and overall sustainability. Among the alternative energy sources, biomass-based electricity through wood chip cogeneration presents a notable improvement in CO_2_ utilization. Co45 at 350 °C reaches a CUF of 210%, capturing more than twice the CO_2_ emitted during the process. Even Fe40, which under conventional grid electricity exhibited limited carbon utilization, improves significantly to 145% CUF, demonstrating the potential of biomass energy to enhance carbon sequestration. However, despite these improvements, the sustainability of biomass cogeneration remains a subject of debate. While it offers a renewable energy alternative, it also introduces critical environmental trade-offs. The combustion of wood chips produces wood ash, which contributes to terrestrial ecotoxicity and eutrophication, while large-scale biomass harvesting risks deforestation, biodiversity loss, and soil degradation. These factors highlight the importance of responsible biomass sourcing and sustainable forest management to mitigate negative externalities. Hydroelectric power stands out as the most sustainable energy option, delivering the highest CUF values across all catalysts. At 350 °C, Co45 reaches 217%, Fe40 achieves 195%, and Fe30 peaks at 225%, indicating a strong carbon-negative potential. The near-zero emissions associated with hydropower eliminate fossil-derived CO_2_ emissions from the energy supply chain, making it the most effective solution for reducing Global Warming Potential (GWP). Unlike biomass, hydroelectricity does not introduce uncertainties related to biogenic carbon accounting or land transformation impacts, further solidifying its position as the optimal energy source for sustainable Fischer–Tropsch synthesis. The role of biogenic carbon accounting is critical when evaluating the sustainability of biomass energy. Unlike fossil fuels, CO_2_ emissions from biomass combustion are theoretically offset by carbon uptake during plant growth. However, the actual neutrality of biomass depends on the rate of forest regrowth, land-use efficiency, and indirect emissions. If biomass is harvested faster than it is replenished, the net CO_2_ balance shifts toward a positive emission scenario, undermining its sustainability benefits. Additionally, land transformation effects, including soil degradation and reductions in long-term carbon stock, can significantly alter the expected carbon sequestration benefits. This is reflected in impact assessment results, where cogeneration exhibits higher terrestrial ecotoxicity and eutrophication levels compared to hydroelectric power. Overall, energy source selection is as critical as catalyst optimization in determining the viability of carbon-neutral Fischer–Tropsch synthesis. Hydroelectric power offers the most effective pathway to achieving net negative CO_2_ emissions while maintaining minimal environmental trade-offs. Biomass cogeneration provides a compelling alternative, significantly improving CO_2_ utilization, but its associated land use and ecosystem impacts necessitate careful sustainability considerations. The findings underscore that even the most efficient catalysts require a complementary low-carbon energy source to maximize their potential for CO_2_ reduction. Integrating high-performance catalysts with renewable energy sources is essential to ensuring that Fischer–Tropsch synthesis serves as a viable long-term solution for carbon-neutral synthetic fuel production while addressing global CO_2_ mitigation goals.

## 4 Conclusion

This study highlights the critical role of catalyst selection, energy sourcing, and process optimization in achieving sustainable Fischer–Tropsch synthesis via CO_2_ hydrogenation, assessed by coupling laboratory performance data with cradle-to-gate life cycle assessment (LCA). A key finding is the trade-off between iron-based (Fe30, Fe40) and cobalt-based (Co45) catalysts in terms of both catalytic performance and environmental impact. Cobalt-based catalysts demonstrate superior CO_2_ conversion efficiencies; however, Co45 presents higher environmental burdens during its production phase, mainly driven by the energy-intensive extraction and processing of cobalt as well as its reliance on critical raw materials with geopolitical and supply risks.^42^ Conversely, the iron-based catalysts are less resource-intensive and more sustainable in terms of material availability, but they exhibit lower overall CO_2_ conversion efficiencies. Fe40, in particular, combines the lowest CO_2_ conversion with the highest impacts per kg of C_2_+ product, reinforcing the importance of optimizing catalyst formulation.

Beyond catalyst selection, the electricity mix strongly affects the results: switching from a fossil-based grid to hydroelectric power substantially improves the environmental profile and can enable net CO_2_-negative operation under the assessed conditions. While these findings provide a strong basis for catalyst selection and process optimization, scaling laboratory data to industrial processes remains a challenge. Laboratory-scale setups typically suffer from higher relative energy consumption and inefficiencies.

Future studies should integrate industrial-scale LCA data, scale-up assumptions and address catalyst circularity to ensure real-world applicability. Ultimately, achieving sustainability in FT synthesis requires a systemic approach, combining high-performance catalysts, low-carbon energy sources, and industrial process optimization, rather than relying solely on catalyst improvements.
